# Supplementary material for: Effects of Ultra-Short Pulsed Electric Field Exposure on Glioblastoma Cells
Source: Int J Mol Sci. 2022 Mar 10;23(6):3001. doi: 10.3390/ijms23063001 (PMC8950115; doi:10.3390/ijms23063001)
Supplement: Supplementary file 1 [file ijms-23-03001-s001.zip › ijms-1604556-supplementary.pdf]

Supplementary Table S1. List of primers used for quantitative real time PCR.

**Human Gene**	**Forward primer**	**Reverse primer**
Cd133	5’- TCCACAGAAATTTACCTACATTGG -3’	5’- CAGCAGAGAGCAGATGACCA -3’
CD15	5’-TGGGCAGGCTGGTCTTGAACT-3’	5’- CACGGCGGCTCACACCTGTA-3’
β III TUB	5’- AGATGTACGAAGACGACGAGGAG-3’	5’- GTATCCCCGAAAATATAAACACAAA-3’
P53	5’- TAACAGTTCCTGCATGGGCGGC-3’	5’-AGGACAGGCACAAACACGCACC-3’
P21	5’- TCC AGC GAC CTT CCT CAT CCA C -3’	5’ -TCCATAGCC TCTACTGCC ACC ATC-3’
Cyclin D1	5’ TGTGCATCTACACCGACAACT-3’	5’- CACAGAGGGCAACGAAGGT-3’
GADD45a	5’ TCGTGAAATGGAAGGGATGG -3’	5’-AGGTTTTGGGCTTGGGTC-3’
CDK2	5’-CGGAGCTTGTTATCGCAAAT -3’	5’ -CACTGGAGGAGAGGGTGAGA -3’
CDK4	5’-CACAGCTGCTGCTGGAAAT-3’	5’-TGCTCACTCCGGATTACCTT-3’
IL6	5 ’-CAAGTCGGAGGCTTAATTACACATG -3 ’	5 ’- AGAAAAGAGTTGTGCAATGGCA -3 ’
CCL20	5’- AAGTTGTCTGTGTGCGCAAATCC-3’	5’- CCATTCCAGAAAAGCCACAGTTTT -3’
iNOS	5’AGTATCACAGGCTTCCATTGACC-3’	5’-GAAGCCGCTGGCATTCCGCA-3’
COX2	5’- CGGTGAAACTCTGGCTAGACAG-3’	5’- GCAAACCGTAGATGCTCAGGGA -3’
β CATENIN	5’-GGTGGACCCCAAGCTTTAG-3’	5’- AGTGGGATGCTGGGTGTAA-3’
SOX 2	5’- GAGCTTTGCAGGAAGTTTGC -3’	5’- GCAAGAAGCCTCTCCTTGAA -3’
NANOG	5’- ACCTTGGCTGCCGTCTCTGG -3’	5’- AGCAAAGCCTCCCAATCCCAAACA -3’
**OCT4**	5’- TTTTGGTACCCCAGGCTATG -3’	5’- TTTTGGTACCCCAGGCTATG -3’
COX2	5’- CGGTGAAACTCTGGCTAGACAG-3’	5’- GCAAACCGTAGATGCTCAGGGA -3’
GADPH	5’- ATTCCACCCATGGCAAATTC -3’	5’- GGGATTTCCATTGATGACAAG -3’
